# Attenuation of the virulence of a recombinant influenza virus expressing the naturally truncated NS gene from an H3N8 equine influenza virus in mice

**DOI:** 10.1186/s13567-016-0400-7

**Published:** 2016-11-15

**Authors:** Woonsung Na, Kwang-Soo Lyoo, Sun-Woo Yoon, Minjoo Yeom, Bokyu Kang, Hyoungjoon Moon, Hye Kwon Kim, Dae Gwin Jeong, Jeong-Ki Kim, Daesub Song

**Affiliations:** 1 College of Pharmacy, Korea University, Sejong, Republic of Korea; 2 Korea Zoonosis Research Institute, Chonbuk National University, Iksan, Republic of Korea; 3 Viral Infectious Disease Research Center, Korea Research Institute of Bioscience and Biotechnology, Daejeon, Republic of Korea; 4 Research Unit, Green Cross Veterinary Products, Yong-in, Republic of Korea

## Abstract

Equine influenza virus (EIV) causes a highly contagious disease in horses and other equids. Recently, we isolated an H3N8 EIV (A/equine/Kyonggi/SA1/2011) from a domestic horse in South Korea that exhibited symptoms of respiratory disease, and found that the EIV strain contained a naturally mutated NS gene segment encoding a truncated NS1 protein. In order to determine whether there was an association between the NS gene truncation and viral virulence, a reverse genetics system was applied to generate various NS gene recombinant viruses using the backbone of the H1N1 A/Puerto Rico/8/1934 (PR/8) virus. In a mouse model, the recombinant PR/8 virus containing the mutated NS gene of the Korean H3N8 EIV strain showed a dramatically reduced virulence: it induced no weight loss, no clinical signs and no histopathological lesions. However, the mice infected with the recombinant viruses with NS genes of PR/8 and H3N8 A/equine/2/Miami/1963 showed severe clinical signs including significant weight loss and 100% mortality. In addition, the levels of the pro-inflammatory cytokines; IL-6, CCL5, and IFN-γ, in the lungs of mice infected with the recombinant viruses expressing a full-length NS1 were significantly higher than those of mice infected with the virus with the NS gene from the Korean H3N8 EIV strain. In this study, our results suggest that the C-terminal moiety of NS1 contains a number of virulence determinants and might be a suitable target for the development of a vaccine candidate against equine influenza.

## Introduction

Equine influenza virus (EIV), which is a member of the genus *Orthomyxovirus*, family *Orthomyxoviridae*, contains a single-stranded, negative-sense RNA in the following eight gene segments: PB2, PB1, PA, HA, NP, NA, M, and NS. This virus is the causative agent of serious respiratory diseases in horses [1]. There are two distinct influenza A virus subtypes in horses; H7N7 and H3N8. Although H7N7 viruses are no longer circulating in horse populations, H3N8 EIV is continuously isolated in Europe, North America, North Africa, and Asia, including South Korea [1, 2]. It was demonstrated that H3N8 EIV was responsible for the emergence of H3N8 canine influenza A virus (CIV) in North America in 2004 [3]. Genetic analysis revealed that H3N8 CIV originated in a racing greyhound that was previously infected with an H3N8 EIV, and this emergent H3N8 CIV, the representative subtype of CIV in North America, is still circulating in pet dogs [3, 4]. Furthermore, two strains of H3N8 EIV were detected in pigs in China [5]. Thus, H3N8 EIV seems to have the potential to cross the host barrier among animal species. Recently, we isolated from a domestic horse in South Korea an H3N8 EIV strain (A/equine/Kyonggi/SA1/2011; KYG11) belonging to the Florida sublineage clade 1 [2]. Interestingly, the KYG11 strain possessed an NS gene segment that harbored a deletion of nucleotides 326 to 348 of NS1 open reading frame, resulting in a frameshift and a premature stop codon. The encoded NS1 protein was 117 amino acids long [2].

The NS gene of influenza viruses encodes two different nonstructural proteins, NS1 and nuclear export protein. The NS1 protein is known to play a role in the enhancement of influenza virus replication by antagonizing interferon (IFN) production in host cells [6, 7]. In contrast, the replication ability of influenza viruses with a truncated NS1 gene is markedly decreased by inhibited IFN-mediated anti-viral responses in host cells [6–8]. Previous studies have shown that a deletion in the NS1 gene may be associated with the virulence of several subtypes of influenza A viruses such as H3N2 and H5N1 in several animal species [9–11].

Therefore, it was hypothesized that the virulence of the Korean H3N8 EIV strain, KYG11, is influenced by the mutated NS gene. In this study, we aimed at elucidating the effect of the 23 nt deletion within the NS gene in vivo and in vitro and used a reverse genetics system to generate recombinant influenza viruses containing the truncated NS gene of KYG11 or different NS genes from other influenza viruses. The NS gene of KYG11 affected the virulence and immune-pathogenic properties of PR/8 virus in vivo and the results provide important insights into this Korean EIV strain, indicating that a specific region of the NS1 gene confers pathogenicity in mice.

## Materials and methods

### Cells

Madin-Darby canine kidney (MDCK, ATCC CRL-2936) cells, human pulmonary epithelial (A549) cells, and human embryonic kidney (293T) cells were obtained from the American Type Culture Collection (ATCC). The MDCK and A549 cells were grown in Dulbecco’s modified Eagle’s medium (DMEM, Gibco, Carlsbad, CA, USA) containing 10% fetal bovine serum (FBS) and antibiotics. The 293T cells were cultured in Opti-MEM (Gibco) supplemented with 5% FBS and antibiotics. All cells were maintained at 37 °C in 5% CO_2_.

### Viruses

The novel H3N8 EIV strain; A/equine/Kyonggi/SA1/2011(KYG11) and H3N8 A/equine/2/Miami/1963 (MA63) were obtained from the ATCC and propagated in the allantoic cavity of 10-day-old embryonated chicken eggs. The eight-plasmid-based reverse genetics system was kindly provided by Dr Richard J. Webby (St. Jude Children’s Research Hospital, TN, USA).

### Generation of recombinant viruses

Viral RNA was extracted from the allantoic fluid using a QIAamp Viral RNA Mini Kit (Qiagen Inc., Valencia, CA, USA). The RNA was reverse transcribed and NS genes were amplified by PCR using a OneStep reverse transcription PCR kit (Qiagen Inc.) with universal primers and then cloned into a pHW2000 plasmid. We used the plasmid-based reverse genetics system to generate the reverse genetics viruses [12, 13].

Briefly, eight pHW2000 plasmids, each containing an individual gene of the eight influenza A virus genes, were transfected into co-cultured MDCK and 293T cells. Recombinant PR/8 (rPR/8) and the following NS-gene reassortant viruses were rescued from the plasmids: PR/8 × NS KYG11 (rPR/8 × KYG^NS^) and PR/8 × NS A/equine/2/Miami/1963 (rPR/8 × MI^NS^). The supernatant collected from transfected cells was used to inoculate 10-day-old embryonated chicken eggs for virus propagation. After the generation of the recombinant viruses, the full genome sequences of the viruses were verified with an RT-PCR and sequencing analysis. Virus stocks were stored at −80 °C until use.

### Western blotting and plaque assay

For immunoblotting, MDCK cells infected with the viruses were lysed in RIPA lysis buffer (1% Triton X-100, 1% deoxycholate, and 0.1% SDS). The proteins were then separated on 10% SDS polyacrylamide gels, then transferred onto a nitrocellulose membrane. The membrane was blocked in 5% skimmed milk buffer, then stained using primary monoclonal mouse antibodies against influenza A NS1 protein (1:2000; sc-130568; Santa Cruz, Dallas, TX, USA) and monoclonal mouse antibodies against influenza A virus NP protein (1:2000; sc-80481; Santa Cruz) at 4 °C overnight. For protein detection, the membrane was incubated with anti-mouse IgG HRP-conjugated secondary antibody and visualized using the ATTO Ez-Capture II system (ATTO, Japan). For the plaque assay, MDCK cells plated in 6-well tissue culture plates were inoculated with tenfold serially diluted viruses. After adsorption for 1 h, the cells were washed and overlaid with 1% low-melting agarose in DMEM containing 2% FBS. After incubation at 37 °C for 72 h, the agarose was gently removed and plaques were visualized with crystal violet staining.

### Virus replication kinetics

To evaluate the kinetics of virus replication in vitro, allantoic fluid containing rPR/8, rPR/8 × KYG^NS^, or rPR/8 × MI^NS^ viruses was harvested, and MDCK and A549 cells were infected with the viruses at a multiplicity of infection (MOI) of 0.01 plaque forming unit per cell. The virus inocula were removed after 1 h. The cells were then washed and infection medium containing 1 μg/mL TPCK-treated trypsin was added. Supernatants were collected 12, 24, 36, 48, and 72 h post-inoculation and stored at −80 °C for titration by TCID_50_ assay. The TCID_50_ was calculated by the method of Reed and Muench [14]. The limit of detection was 10 TCID_50_/mL. In addition, the kinetics of the harvested viruses was determined by inoculating 10-day-old embryonated chicken eggs with serial dilutions of viruses. The limit of detection of the assay was 10 EID_50_/mL.

### Mouse experiments

All animal experiments were performed in biosafety-level-2 facilities at the Korean Research Institute of Bioscience and Biotechnology (Daejeon, South Korea). We followed the General Animal Care Guideline as required by the Institutional Animal Care and Use Committee of the institute (Approval Number: # KRIBB-ACE-14096).

To determine the 50% mouse lethal dose (MLD_50_) of each virus, 6-week-old C57BL/6 mice (Korea Animal Technology, Pyeongtaek, South Korea) were inoculated intranasally with tenfold serial dilutions containing 10^2^ to 10^8^ EID_50_ of each virus in a 30 μL volume. The MLD_50_ was calculated using the method of Reed and Muench. To compare the virulence of the rPR/8, rPR/8 × KYG^NS^, and rPR/8 × MI^NS^ viruses, ten mice were used to monitor the survival rates and body weights, and another twelve mice were used for lung titration of each recombinant virus strain. The anesthetized mice were inoculated intranasally with 300 EID_50_ (in a 30 μL volume) of each virus into a single nostril. For the mock control group, ten and twelve mice inoculated with phosphate-buffered saline (PBS) were used for body weight measurement and lung titration, respectively.

For virus titration in the lungs, three mice in the infection group were sacrificed at 3, 5, 7, and 9 days post-infection (dpi), and lung tissues were collected and homogenized in 1 mL of PBS with antibiotics. The supernatant collected from the homogenized tissue was titrated in eggs. All mice were monitored daily for 14 days for weight changes and mortality, and mice that lost more than 25% of their body weight were euthanized.

### Histopathological examination

Lung tissue samples were harvested from the mice on 7 dpi. The samples were collected in 10% buffered formalin to fix the tissues and were then embedded in paraffin wax, sectioned (4–5 μm thick sections), and placed on glass slides. For the histological examination of the tissues, they were stained with hematoxylin and eosin (H&E) and examined by a pathologist.

### Cytokine assay

The cytokine level in bronchoalveolar lavage (BAL) fluid was determined using a Bio-Plex Pro™ cytokine assay kit (Bio-Rad, Hercules, CA, USA). The pro-inflammatory cytokines: interleukin (IL)-6, interferon-gamma (IFN-γ, and CCL5 (RANTES; regulated upon activation normal T cell expressed and presumably secreted) were measured according to the manufacturer’s instructions. Briefly, 50 μL BAL fluid samples were incubated with antibody-coupled beads. These immune complexes were washed and incubated first with a biotinylated detection antibody and then with streptavidin–phycoerythrin prior to assessing the cytokine concentration. The pro-inflammatory cytokine levels were determined using a Bio-Plex 200 System multiplex array reader (Bio-Rad) using software provided by the manufacturer (Bio-Plex Manager Software 4.1.1).

### Statistical analysis

The data for body weight changes, viral titers, and levels of the pro-inflammatory cytokines in the lungs of the mice were analyzed by the two-way ANOVA with Bonferroni post-tests. Statistical analyses were performed using GraphPad Prism version 5 (GraphPad Software, La Jolla, CA, USA). A *P* value less than 0.05 was considered statistically significant.

## Results

### Characterization of recombinant viruses

**Figure 1:**
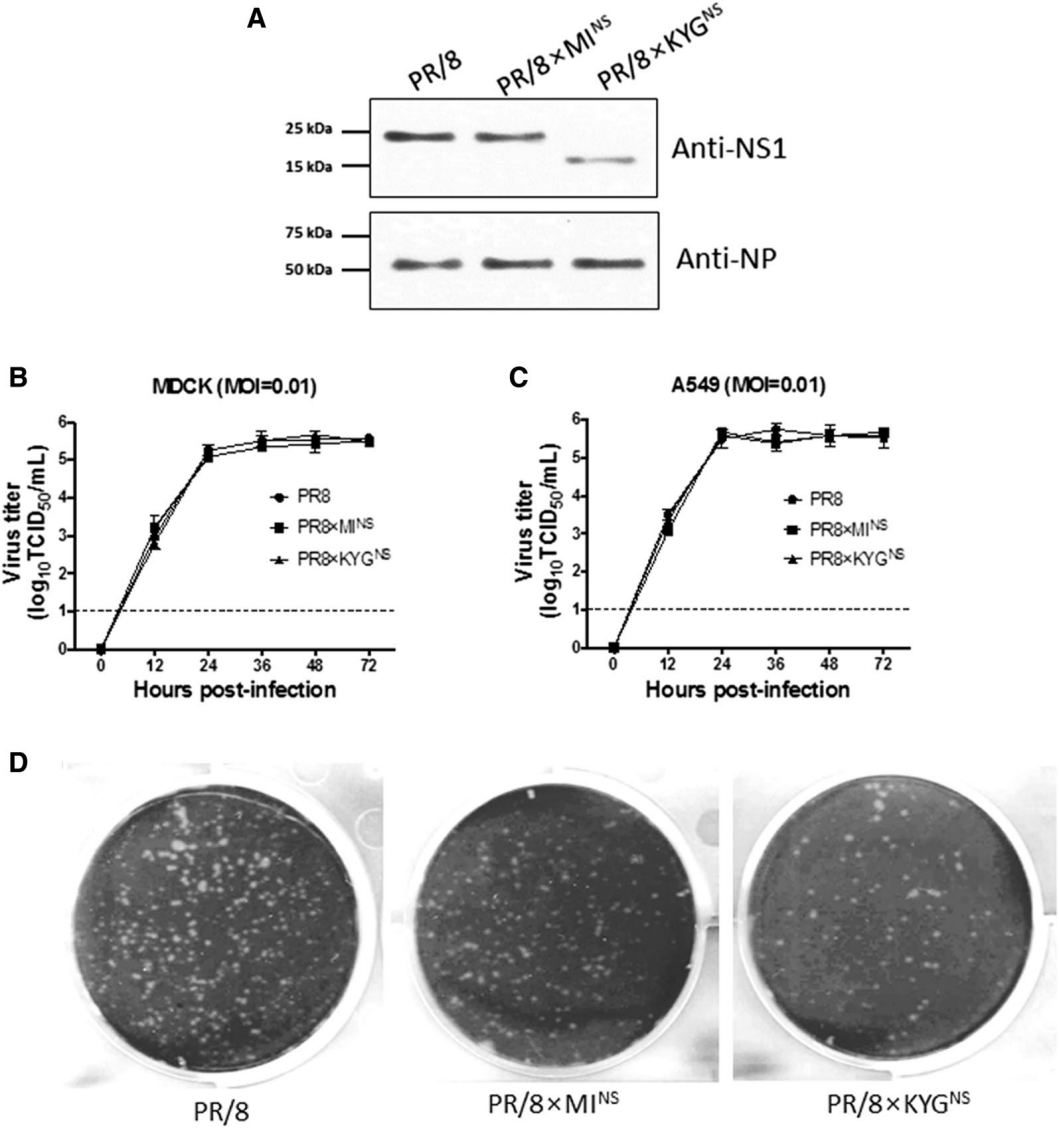
**Western blot analysis, growth kinetics, and plaque phenotyping of the recombinant viruses.** Immunoblot of the NS1 protein in extracts from MDCK cells infected with PR/8, PR/8 × KYG^NS^, and PR/8 × MI^NS^ viruses at an MOI of 1 for 12 h (**A**). The protein was detected using a mouse anti-NS1 primary antibody, and the molecular weight in kDa is shown on the left of the membrane. MDCK (**B**) and A549 (**C**) cells were infected with the PR/8, PR/8 × KYG^NS^, or PR/8 × MI^NS^ virus at an MOI of 0.01, and the virus was titrated in the supernatant that was collected at the indicated time points. The detection limit was 1 log_10_ TCID_50_/mL (dotted line). Data are shown as the mean ± standard deviation from three independent experiments. Plaque assay performed with PR/8, PR/8 × KYG^NS^, and PR/8 × MI^NS^ viruses (**D**). The tissue culture plates infected with the viruses were fixed and stained with crystal violet dye.

To determine the functionality of the deleted nucleotides in the truncated NS gene, we successfully generated reverse genetics viruses; rPR/8, rPR/8 × MI^NS^; and rPR/8 × KYG^NS^. As a first attempt to characterize the three recombinant viruses, their sequences were checked, and we confirmed that cells infected with the recombinant virus rPR/8 × KYG^NS^ virus expressed a ~15 kDa NS1; however full-length NS1protein of approximately 26 kDa was revealed for the rPR/8 and rPR/8 × MI^NS^ viruses by western blotting (Figure 1A).

The growth properties of the three viruses were determined in embryonated chicken eggs. All of the reverse genetics viruses grew to high titers in eggs (Table 1), with endpoint titers after a single egg passage reaching 10^7.9^ EID_50_/mL for rPR/8, 10^8.1^ EID_50_/mL for rPR/8 × MI^NS^, and 10^8.3^ EID_50_/mL for rPR/8 × KYG^NS^.

**Table 1:** **Characteristics of the recombinant viruses**

Virus	Virus titer (EID_50_)^a^	MLD_50_ (EID_50_)^b^
PR/8	10^7.9^	10^3.4^
PR/8 × MI^NS^	10^8.1^	10^4.3^
PR/8 × KYG^NS^	10^8.3^	10^7.8^

Virus titer in embryonated eggs, MLD_50_ (expressed in EID_50_ units).

MLD: mouse lethal dose, EID: egg infectious dose.

^a^The EID_50_ was calculated by the Reed and Muench method.

^b^N: 10 for each virus infection group.

Next, how the truncated NS gene affected the growth kinetics of the viruses was determined in vitro. We infected MDCK and A549 cells with the rPR/8, rPR/8 × KYG^NS^ and rPR/8 × MINS viruses at an MOI of 0.01 and observed their growth kinetics for 72 h. We found that all recombinant viruses grow to a similar titer in both cell lines at each time point, indicating that the truncated NS gene did not substantially affect the replicative capacity of these viruses in cell culture (Figures 1B and C). In addition, all three viruses displayed the same plaque phenotype at 37 °C (Figure 1D).

### Virulence of recombinant viruses in mice

To evaluate how the truncated NS gene affected virulence in vivo, we inoculated mice with the recombinant viruses. The rPR/8 and rPR/8 × MI^NS^ viruses showed comparable virulence, with MLD_50_ of 10^4.25^ and 10^3.92^ EID_50_, respectively, whereas the rPR/8 × KYG^NS^ recombinant showed significant attenuation, with an MLD_50_ value of 10^7.75^ (Table 1). To further investigate the virulence of these viruses in mice, we inoculated mice with 300 EID_50_ (in a 30 μL volume) of each recombinant virus and evaluated clinical signs, mortality, weight loss, and viral load in the lungs. Virus replication kinetics in the lung was determined by measuring virus titers at 3, 5, 7, and 9 dpi.

**Figure 2:**
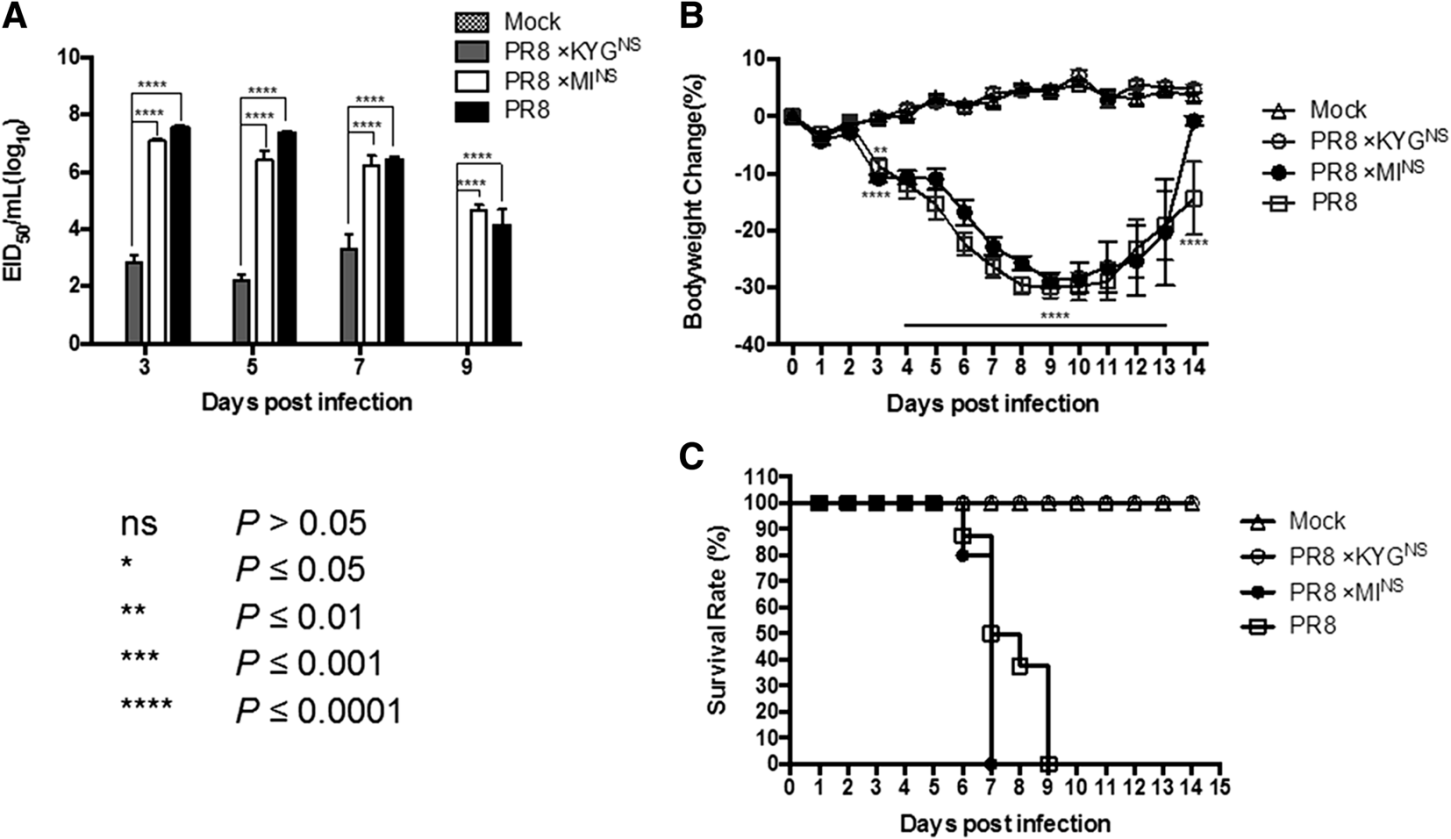
**Virulence of the recombinant viruses.** The anesthetized 6-week-old C57BL/6 mice were inoculated intranasally with 300 EID_50_ (in a 30 μL volume) of PR/8, PR/8 × KYG^NS^, or PR/8 × MI^NS^ virus into a single nostril. Viral lung titers were measured at 3, 5, 7, and 9 dpi (**A**), and body weight changes were monitored daily for 14 days (**B**). Mortality was measured by defining an endpoint of 25% body weight loss (**C**). Asterisks indicate statistically significant differences between groups (*P ≤ 0.05; **P ≤ 0.01; ***P ≤ 0.001; and ****P ≤ 0.0001).

The virus titers in mice infected with rPR/8 × KYG^NS^ were at least one hundred to one thousand fold lower than the virus loads in the lungs of mice inoculated with the two other viruses (Figure 2A). The body weights of mice inoculated with rPR/8 × KYG^NS^ gradually increased from 1 to 14 dpi. In contrast, there was rapid and dramatic weight loss of over 25% in the mice infected with rPR/8 or rPR/8 × MI^NS^ (Figure 2B). The mice infected with rPR/8 × KYG^NS^ exhibited no clinical signs and showed 100% survival. In contrast, all mice infected with rPR/8 or rPR/8 × MI^NS^ exhibited progressive signs such as inactivity, ruffled fur, lack of appetite, hunched backs, and labored breathing (Figure 2C).

### Pulmonary histopathology in mice inoculated with recombinant viruses

**Figure 3:**
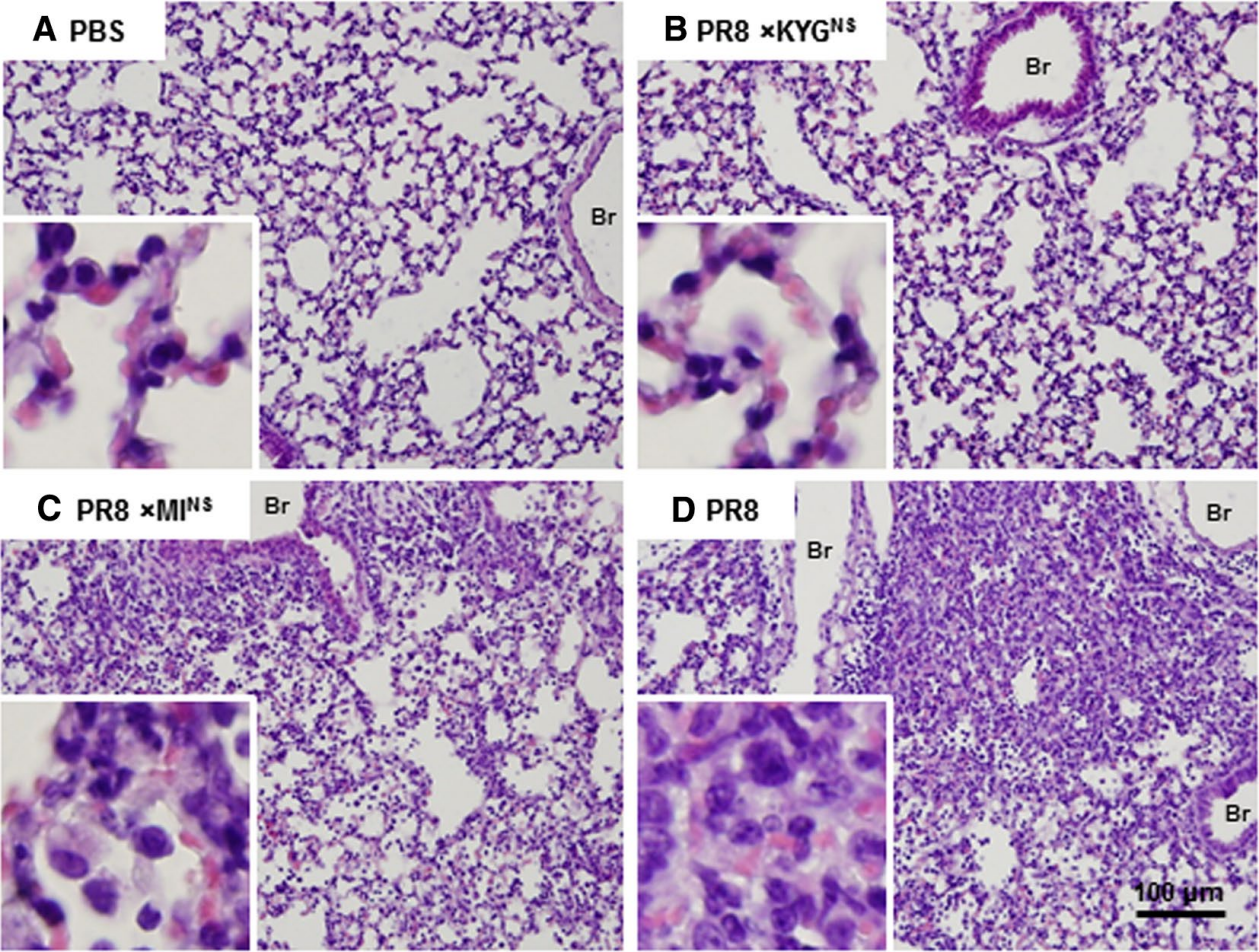
**Lung histopathology of mice infected with the recombinant viruses.** Histopathology of lungs from mice infected with each PR/8 virus containing a different NS gene from a different influenza virus. Mice were inoculated intranasally with each recombinant virus at a dose of 300 EID_50_ (in a 30 μL volume), and lung tissues were collected on day 7 following virus inoculation. Images are representative of three mice per group (**A** mock-infected; **B** PR/8 × KYG^NS^—infected; **C** PR/8 × MI^NS^—infected; and **D** PR/8-infected). The bar represents 100 μm. Images were obtained at 20× magnification.

To examine viral pathology, we performed H&E staining on lung tissues collected on days 7 post-infection from three mice. Mice infected with PBS or rPR/8 × KYG^NS^ showed normal lung histology characterized by well-preserved pulmonary alveoli and bronchioles, whereas those infected with rPR/8 × MI^NS^ exhibited mild interstitial and suppurative pneumonia characterized by slightly thickened alveolar septa, with moderate hyperplasia of type II pneumonocytes and infiltration of a small number of mononuclear cells in the alveoli and alveolar septa. Mice infected with rPR/8 exhibited interstitial and suppurative pneumonia characterized by disrupted lung parenchyma, notably thickened alveolar septa due to type II alveolar cell hyperplasia, and marked infiltration of lymphocytes and mononuclear cells in the alveoli and interstitium (Figure 3).

### Induction of cytokine expression

**Figure 4:**
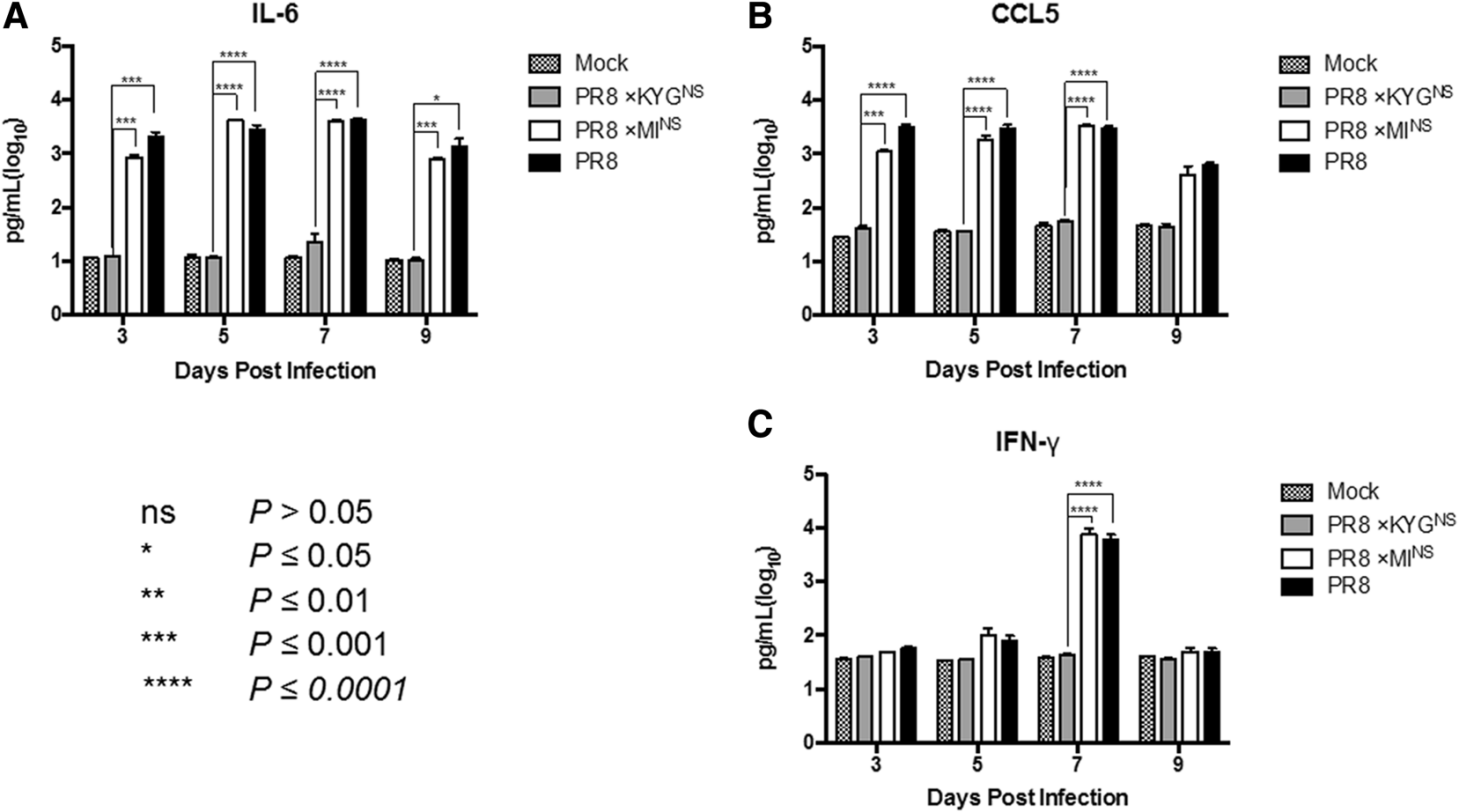
**Cytokine levels in mice infected with the recombinant viruses.** The concentrations of induced cytokines and chemokines were determined in the infected mice (*n* = 3). Levels of (**A**) IL-6, (**B**) CCL5, and (**C**) IFN-γ were measured in bronchoalveolar lavage fluids harvested at 3, 5, 7, and 9 dpi. Asterisks indicate statistically significant differences between groups (*P ≤ 0.05; **P ≤ 0.01; ***P ≤ 0.001; and ****P ≤ 0.0001).

To analyze pro-inflammatory cytokine expression in mice infected with the different NS-expressing viruses, the BAL fluid from infected mice was collected at 3, 5, 7, and 9 dpi, and secreted cytokines and chemokines were measured using a Micro Bead Suspension Array. The levels of IL-6, which stimulates an immune response, and CCL5, which plays an active role in recruiting leukocytes into the site of inflammation increased by about 100-fold (IL-6) or >tenfold (CCL5) after rPR/8 and rPR/8 × MI^NS^ virus infection, whereas the rPR/8 × KYG^NS^ infection group showed no significant increase in the levels of these two pro-inflammatory cytokines (Figures 4A and B). The results for IFN-γ were similar: the level of this cytokine in the rPR/8 and rPR/8 × MI^NS^ infection groups was significantly higher than that of the rPR/8 × KYG^NS^ group, especially at 7 dpi (Figure 4C).

## Discussion

The NS1 protein is generally regarded as the primary factor by which influenza A viruses antagonize the host INF response to enhance viral replication [15]. It has been suggested that the protein inhibits the polyadenylation of cellular mRNA and promotes the addition of poly A tails onto viral mRNA and sequesters viral dsRNA, a replicative intermediate, inactivating cellular protein kinase R (PKR) and suppressing the IFN response [15, 16].

**Figure 5:**
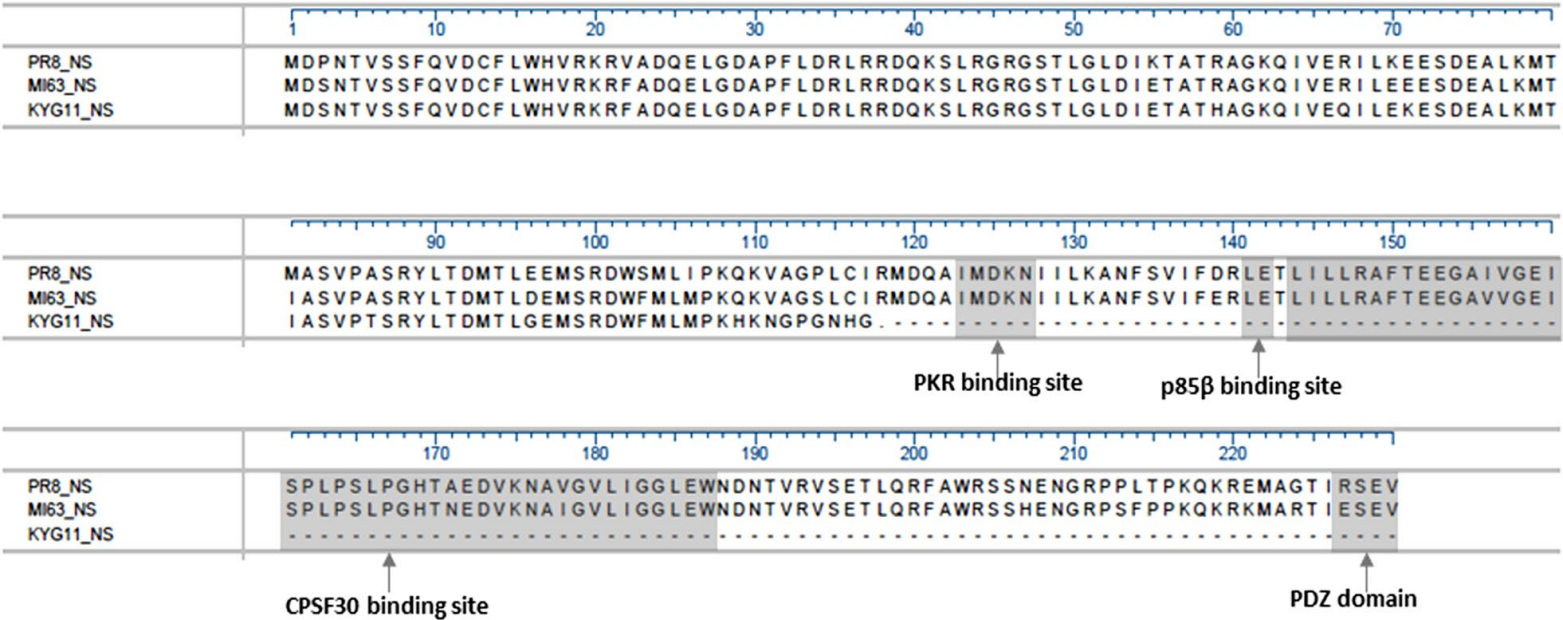
**The alignment of the amino acid sequences of the NS1 gene from KYG11, MA63, and PR/8 viruses.** The 23-nt deletion results in a frameshift, generating a premature stop codon and a truncated NS1 protein.

The inhibition of PKR, which plays a critical role in host antiviral responses mediated by IFN, occurs through the direct binding of the NS1 protein of influenza A virus to PKR. It has been shown that PKR activation is blocked by direct binding to the amino acid (AA) residues 123–127 of NS1 [17]. Furthermore, p85β, a regulatory subunit protein of phosphatidylinositol-3-kinase (PI3 K), which inhibits apoptosis to ensure viral replication, activates PI3K signaling [18]. For this pathway, the binding motifs located at AA 164–167 and AA 141–142 in NS1 are essential for mediating the interaction between NS1 and p85β [18]. PDZ domain-containing proteins may provide cell signaling and polarity functions in transduction pathways, and the binding motif consists of the last four C-terminal AA residues of NS1 [19]. In addition, the NS1 protein binds to the 30-kDa subunit of the cleavage and polyadenylation specificity factor (CPSF30) at residues AA 144–186 of NS1 and inhibits the 3′- end processing of cellular pre-mRNA and then facilitates efficient influenza A virus replication [20]. The alignment of NS1 gene AA sequences from KYG11, MA63, and PR/8 viruses is presented in Figure 5. Consequently, for the KGY11 strain, the binding sites associated with the cell signaling pathway for the antiviral response were lacking because of the premature termination of the NS1 protein.

In particular, horses vaccinated with EIV with a C-terminally truncated NS1 gene showed protection effects and significantly reduced clinical signs in response to challenge with wild-type EIV [21]. Furthermore, influenza virus with C-terminal NS1 truncations was characterized by not only a high degree of attenuation in vitro but also a higher replication capacity and stronger IFN stimulation in vivo compared to NS1-deleted virus in vivo [22, 23]. Accordingly, it is hypothesized that EIV with the C-terminally truncated NS1 gene presents a unique advantage for the development of an EIV vaccine.

For these reasons, we hypothesized that the truncated NS gene could affect the replication of the influenza virus, and we established a reverse genetics system to characterize this effect in vitro and in vivo. Here, we report the extensive characterization of KYG11, an EIV strain isolated from a horse that possesses a unique naturally mutated NS gene segment encoding a truncated NS1 protein. The truncation did not affect the viral replication of rPR/8 in either MDCK or A549 cells; rPR/8 × KYG^NS^ showed viral titers comparable to those of rPR/8 and rPR/8 × MI^NS^ in both cell lines. This finding was in accordance with a previously reported in vitro characterization of influenza viruses containing an NS gene segment with a long deletion [24]. Nevertheless, the finding of no difference in growth kinetics between PR8-based recombinant viruses containing NS1 from KYG11 and MA63 in vitro remains controversial because both viruses previously showed notable differences in growth kinetics in MDCK cell culture [2]. Probably, there are mechanisms other than those involving the NS1 protein used to evade the antiviral effects of interferon in cells, and several factors may contribute to the growth efficiency of influenza virus, in particular PR/8, although the NS1 protein is thought to play a significant role in virus replication [22]. Accordingly, the functional roles of viral factors that affect growth characteristics need to be further characterized.

However, the NS gene of the KYG11 strain when inserted into the PR/8 background conferred dramatically attenuated pathogenicity in mice. Higher viral loads in the lungs, significant weight loss, and 100% mortality following infection with the rPR/8 or rPR/8 × MI^NS^ virus were observed in mice, in contrast to what was seen in rPR/8 × KYG^NS^ infections with an equivalent dose. To explain the possible association between reduced pathogenicity and the truncated NS gene, pro-inflammatory cytokines in the lungs of infected mice were measured. In assays for a panel of cytokines, infection with rPR/8 × KYG^NS^ did not induce overexpression of pro-inflammatory cytokines (IL-6, CCL5, and IFN-γ) on the contrary to what was observed with the two other viruses.

In human volunteers infected with influenza viruses, increased IL6 levels were associated with rapid activation of the innate immune response and pathological signs [25, 26]. Furthermore, IL-6 was strongly upregulated in ferrets infected with virulent strains of H1N1 and H3N2 [27]. CCL5 is a potent chemotactic cytokine that recruits Th1 and Th2 pro-inflammatory cells and contributes to immunopathology by activating cytotoxic T lymphocytes in the lungs following influenza virus infection [28, 29]. IFN-γ is a pro-inflammatory cytokine with immune regulatory activities; it also plays a role in the activation of macrophages and differentiation of Th1 cells from T cells. Increased levels of IFN-γ in response to influenza virus infection expand the virus-specific T cell population and enhance the natural killer cell response [30–32].

In this study, the elevated levels of three pro-inflammatory cytokines may have contributed to the prominent infiltration of lymphocytes and suppurative pneumonia in mice infected with rPR/8 or rPR/8 × MI^NS^, whereas the normal lungs of the rPR/8 × KYG^NS^-infected mice may be associated with the virtually unchanged expression of the pro-inflammatory cytokine expression. A similar finding regarding pro-inflammatory cytokines and lung pathology associated with an H5N1 influenza virus NS gene with a deletion was previously reported [11]. This group also found that a PR/8 influenza virus engineered to incorporate the NS gene of a low pathogenic virus showed attenuated pathogenicity in mice, characterized by reduced production of pro-inflammatory cytokines [11]. Mutations or deletions within the NS1 gene have been shown to affect the ability of influenza viruses to antagonize IFN production and to alter the virulence of influenza viruses in different hosts [11, 15, 33, 34].

In this study, an attenuation of the virulence in mice of recombinant PR/8 containing the truncated NS gene segment (a nucleotide deletion from position 326 to 348) derived from H3N8 EIV was observed. Our results suggest that the C-terminal moiety of NS1 harbors virulence determinants for influenza viruses and therefore might be an appropriate target for the development of antiviral drugs and vaccine candidates against equine influenza.
